# Validation of Verbal Autopsy Tool for Ascertaining the Causes of Stillbirth

**DOI:** 10.1371/journal.pone.0076933

**Published:** 2013-10-09

**Authors:** Sidrah Nausheen, Sajid B. Soofi, Kamran Sadiq, Atif Habib, Ali Turab, Zahid Memon, M. Imran Khan, Zamir Suhag, Zaid Bhatti, Imran Ahmed, Rajiv Bahl, Shireen Bhutta, Zulfiqar A. Bhutta

**Affiliations:** 1 Division of Women & Child Health, Aga Khan University, Karachi, Pakistan; 2 Maternal and Newborn Health Program, Research & Advocacy Fund, Islamabad, Pakistan; 3 Department of Child and Adolescent Health and Development, World Health Organization, Geneva, Switzerland; 4 International Vaccine Institute, Seoul, Korea; 5 Department of Obstetrics & Gynecology, Jinnah Postgraduate Medical Center, Karachi, Pakistan; 6 Centre of Excellence in Women & Child Health, Aga Khan University, Karachi, Pakistan; 7 Center for Global Child Health, Hospital for Sick Children, Toronto, Canada; University of Alabama at Birmingham, United States of America

## Abstract

**Objective:**

To assess performance of the WHO revised verbal autopsy tool for ascertaining the causes of still birth in comparison with reference standard cause of death ascertained by standardized clinical and supportive data.

**Methods:**

All stillbirths at a tertiary hospital in Karachi, Pakistan were prospectively recruited into study from August 2006- February 2008. The reference standard cause of death was established by two senior obstetricians within 48 hours using the ICD coding system. Verbal autopsy interviews using modified WHO tool were conducted by trained health workers within 2- 6 weeks of still birth and the cause of death was assigned by second panel of obstetricians. The performance was assessed in terms of sensitivity, specificity and Kappa.

**Results:**

There were 204 still births. Of these, 80.8% of antepartum and 50.5% of intrapartum deaths were correctly diagnosed by verbal autopsy. Sensitivity of verbal autopsy was highest 68.4%, (95%CI: 46-84.6) for congenital malformation followed by obstetric complication 57.6%, (95%CI: 25-84.2). The specificity for all major causes was greater than 90%. The level of agreement was high (kappa=0.72) for anomalies and moderate (k=0.4) for all major causes of still birth, except asphyxia.

**Conclusion:**

Our results suggest that verbal autopsy has reasonable validity in identifying and discriminating between causes of stillbirth in Pakistan. On the basis of these findings, we feel it has a place in resource constrained areas to inform strategic planning and mobilization of resources to attain Millennium Development Goals.

## Introduction

Every year 3.2 million babies are still born, with no signs of life and about 3.3 million babies die in neonatal period worldwide [1,2] yet stillbirths are given far less attention than it deserves. A bulk of these stillbirths (an estimated 98%) occurs in resource constrained countries of sub Saharan Africa and south Asia resulting mostly from antepartum or intra partum complications [3]. Most of the still births in these areas occur at home and thus remain un-notified and un-registered. The dearth of data thwarts perinatal health planning as it depends upon the availability of accurate data [4]. The need for collecting accurate information about the timing, causes and the burden of stillbirths and early neonatal deaths is therefore critical for maternal and child health care planning.

The focus of global attention has long been on the intrapartum and immediate postnatal period [5] with still birth receiving less prominence in global, international health policy. The reported still birth incidence in south Asia is 32/1000 births which is critical [2] and about similar number is unreported, but without knowledge of the underlying causes and without addressing them in health policies Millennium Development Goals (MDGs) is a far cry from reality.

In the absence of a comprehensive registration system, verbal autopsy is the only tool for gathering cause specific mortality data from the community. Verbal autopsy is an indirect method of ascertaining the cause of death from information about symptoms, signs and circumstances preceding death, obtained from the caretakers of the deceased. Global perinatal and neonatal mortality rates are emerging from Asia [6-8] and Africa yet most of these studies have small sample size, do not use standardized tool and are based in health centers [9]. Therefore there is a need for validation of the verbal autopsy tool so that it can be used in community based studies in other resource constrained settings.

Verbal autopsy has been used extensively over years for ascertaining the cause of child death [10]. Validation studies have shown reasonable sensitivity and specificity of childhood verbal autopsy for major causes of childhood death in comparison with physician’s certification of death [11,12] but have shown poor diagnostic accuracy for establishing causes of neonatal death [6]. The childhood verbal autopsy was thus revised by an informal group of WHO in 2002 to include specific modules for still birth and neonatal death [13]. Over last few years this revised VA has been used in studies [6] but its performance has not been systemically assessed. 

Our study aimed at validating the performance of a standardized verbal autopsy tool in estimating cause specific mortality for major causes of neonatal death and stillbirths. The objective of this paper is to assess the sensitivity, specificity and level of agreement of revised WHO verbal autopsy in ascertaining the cause specific mortality fractions (CSMF) for major causes of stillbirth in comparison with a reference standard cause of death ascertained by standardized clinical and supportive radiology and laboratory data collected prospectively in the hospitals. Validation for neonatal mortality is discussed in another comparison paper although data was collected contemporaneously.

## DESIGN AND METHODOLOGY

### Methods

**Figure 1:**
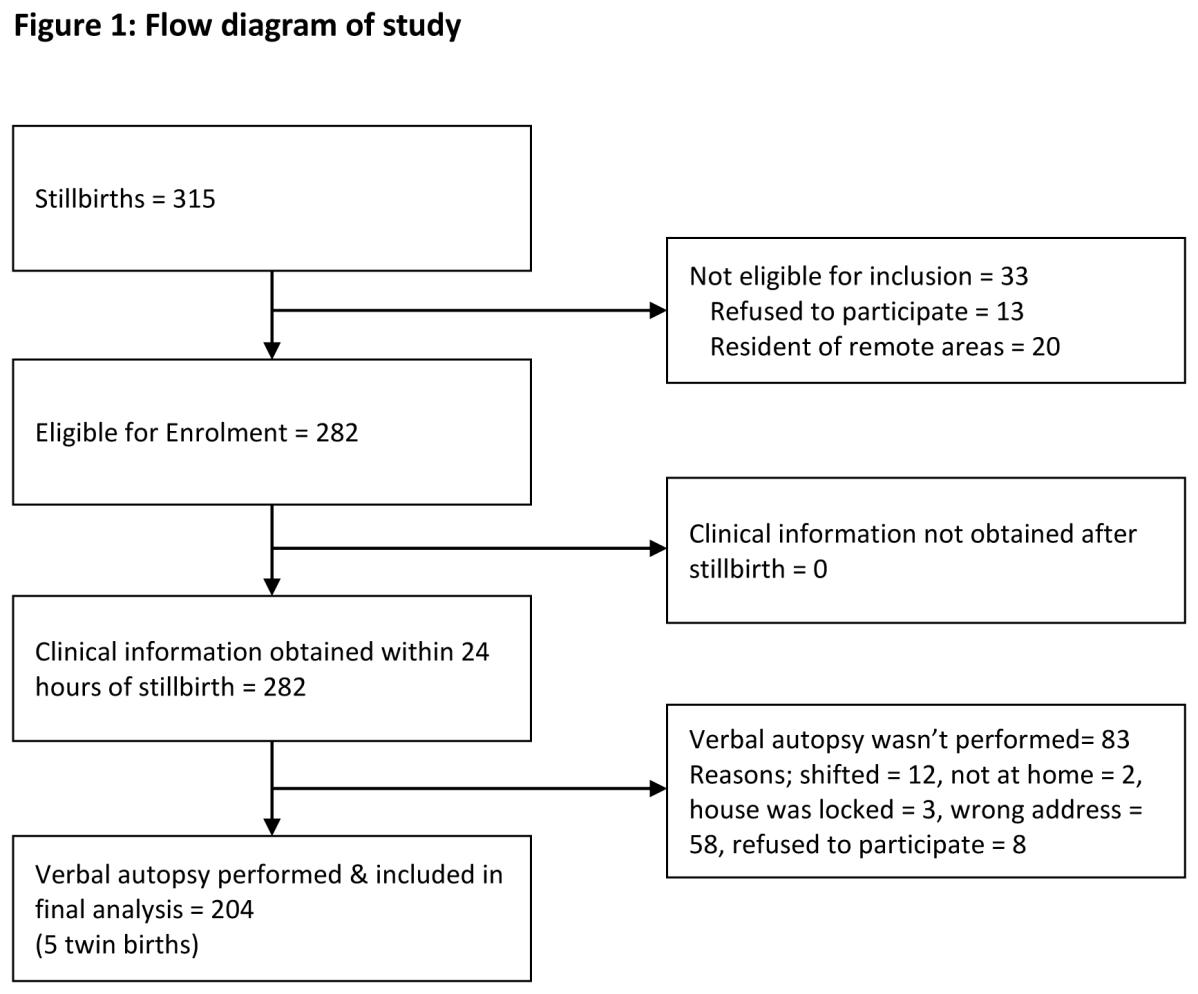
Flow diagram of study.

The study was conducted in two major cities of Pakistan, Karachi and Hyderabad with collaboration of WHO in three tertiary care hospitals. The stillbirths (the subjects in this study) were recruited only from OBS/GYN unit of Jinnah Postgraduate Medical Center, Karachi whereas neonatal deaths were recruited from Civil Hospital Hyderabad and National Institute of Child Health, Karachi. Data was prospectively collected from August 2006 to February 2008 over 18 month. All stillbirths admitted in the hospital during the study period to mothers, who resided within 100 kilometers of the hospital, consented to be included in study, and were more than 28 weeks pregnant were included. Additionally, assignment of cause of death within 48 hours of stillbirth was another criterion for inclusion. Total 315 stillbirths were admitted to hospital in study period, 20 of them resided in remote areas and did not met the inclusion criteria whereas 13 refused to participate. Thus 282 cases were recruited form the hospital. Verbal autopsy could not be performed in 83 cases “Figure 1” of which 58 gave wrong address, 8 refused to participate, 12 shifted their home and 5 were not at home. Thus 204 cases including 5 sets of twin babies were included in final analyses. The hospital record information was considered as reference data and verbal autopsy data (verbatim) from community was used as study data.

### Ascertaining the Reference Cause of Death- Hospital Records

 Details of the events around stillbirths that took place in the hospital were recorded by two trained medical officers (graduates). The medical history, problems in previous pregnancy, antenatal care, complications of pregnancy and labor were recorded on a standardized labor delivery record form. All available laboratories & other investigations were also recorded in this form. Besides that addresses and contact details were also recorded to conduct verbal autopsy. The forms were then checked for completeness and errors by supervisors.

**Figure 2:**
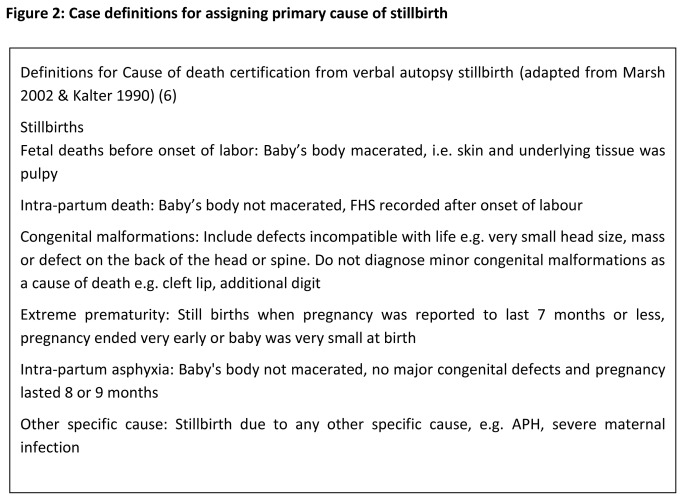
Case definitions for assigning primary cause of stillbirth.

**Figure 3:**
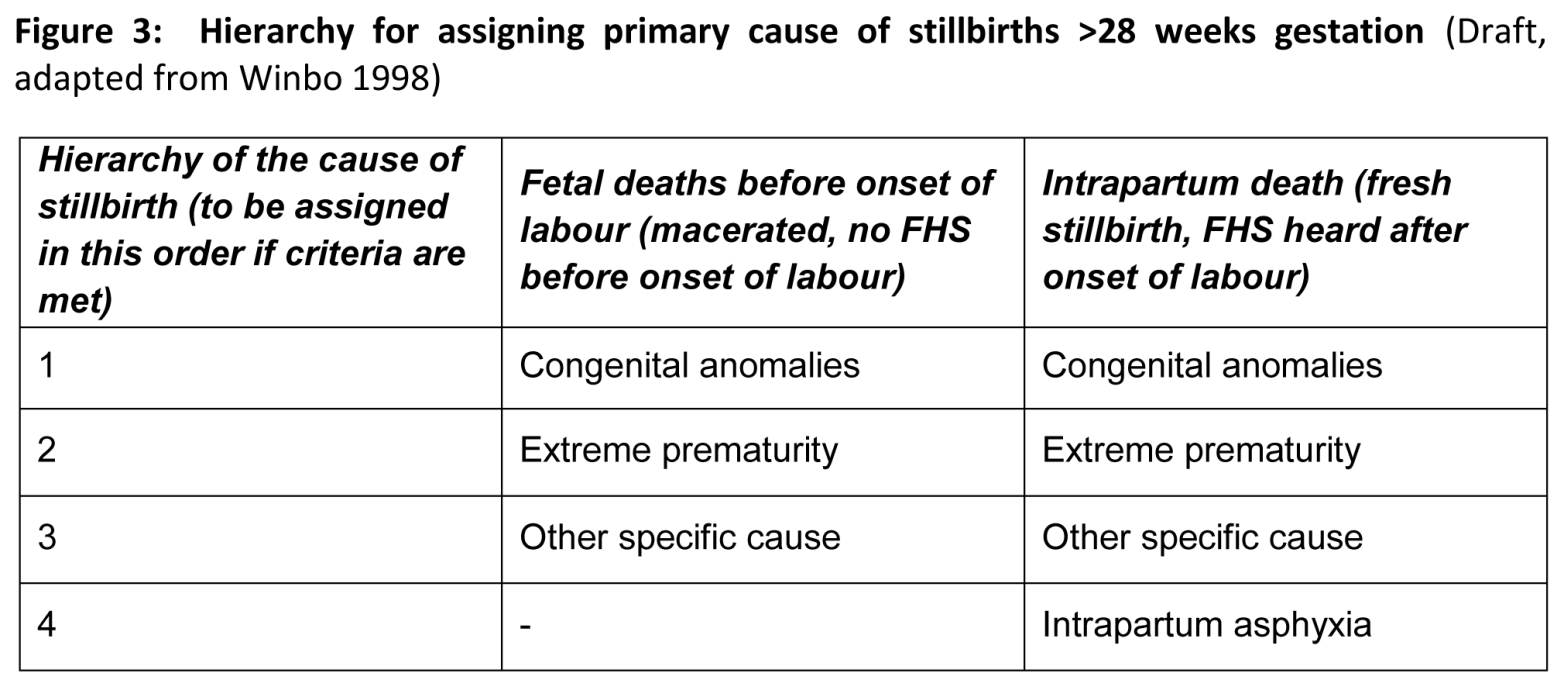
Hierarchy for assigning primary cause of stillbirths >28 weeks gestation (Draft, adapted from Winbo 1998).

Two qualified (FCPS) expert Obstetricians with more than 10 years of clinical experience reviewed the available information, hospital records (history, lab investigation, death certificate, radiological evidence) for all stillbirths and assigned a reference standard primary cause of stillbirth in the light of *International statistical classification of diseases and related health problems, 10th revision* (ICD-10) They received extensive training in 3 days workshop before starting the study on methodology and cause assignment. They were kept independent and blinded to each other in determining the cause of death. In order to standardize the assignment of primary cause of death, a standardized instruction manual for guiding physicians in the assignment of cause of death was developed and used across the study sites. The manual provided information on the process of assigning a cause of death, including ascertainment of adequacy of information, case definitions, “Figure 2” list of causes of death, and the hierarchy of causes of death .The hierarchy of cause of death was adapted from Neonatal and Intrauterine Death Classification according to Etiology, NICE [15], “Figure 3”. The purpose was to assign a single primary cause of death as deceased may have more than one causes. Events that took place first, are placed higher in the hierarchy than events happening later. For example, if a still born with a lethal congenital anomaly was born premature, the cause of death was recorded as congenital anomaly. 

If the two Obstetricians failed to agree, the record was reviewed by a third senior obstetrician with clinical experience of 20 years, and the cause on which two of the three agreed was assigned. If all three did not agree on single cause it was labeled as “unclassifiable”. We did not have data from autopsy or placental histology.

### Assignment of cause of death from Verbal autopsy

The verbal autopsy instrument modified from the WHO/LSTMH/JHU instrument for the evaluation of stillbirth & neonatal deaths (2000) was used. It was modified slightly to adjust cultural sensitivity and norms and also excluded irrelevant questions according to our study objectives. This questionnaire has different sections for basic information about the deceased neonate and still birth and included both narrative and close ended questions. Instrument was translated into local language Sindhi and back translated in English to ensure content validity. Pretesting of the instrument was also performed to identify the problems that could encounter during instrument administration and drive possible solutions. The verbal autopsy interviews were performed between 2 to 6 weeks after death using the standard questionnaire for stillbirths. A well-trained non-medical female interviewer, with an education level ranging from high school to college graduate conducted the verbal autopsy at home. The mother was the primary respondent; in case of recall bias a female relative present at birth/during illness was asked to assist. However, the health care provider who attended the birth was not interviewed for the verbal autopsy. If the respondent was not available on the first visit, one repeat visit was made to find respondent. Written informed consent in the local language was obtained. During the interview, pictorials of major congenital malformations, low birth weight were shown to aid recall. 

**Figure 4:**
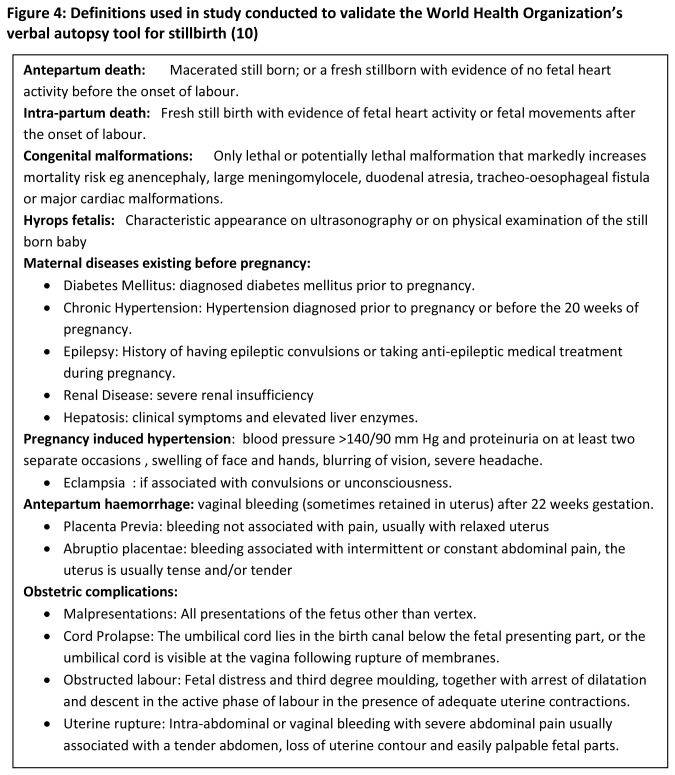
Definitions used in study conducted to validate the World Health Organization’s verbal autopsy tool for stillbirth (10).

Assignment of cause of death in the light of given standardized case definition and list of causes of still births “Figure 4” based on those developed by WHO in 2000 [10] was performed by an independent review of the completed verbal autopsy questionnaires by second panel of two expert obstetricians whose experience was similar to the obstetricians working in study hospital. These experts were kept blinded to the clinical information and to the hospital-based, reference- cause of death. When there is disagreement between the two, a third obstetrician reviewed the same case and the cause on which two of the three agreed was assigned. If all three did not agree on single cause it was labeled as “unclassifiable”. Primary and secondary associated causes of stillbirths were coded; primary cause of death was analyzed.

### Training

A six day’s training workshop was organized to train the community health workers for verbal autopsy interview and recording of information on the instrument. The training focused on the interviewing techniques, and the concepts used in the instrument. Objectives of the study and underlying meaning of the questions used in questionnaire were elaborated in a class room presentation, small and large group discussions. Audio visual aids were also used as per need. Simulated interview were conducted for practice followed by mock interviews at field site being closely observed by one of the investigators. Feedbacks were given to trainees.

Project medical officers (already working as postgraduate students in same hospital) were trained for three days, in recording information about stillbirth from case files in the hospital on a standardized form. 

A 3 days orientation training was arranged for the team of obstetricians to record cause of death in accordance with ICD coding system 10th version and assigning primary single cause as per hierarchy by NICE [15] given in the manual. Both groups received similar training regarding case definitions, list of causes of death and its hierarchy and were explained about utilization of hospital reviews and verbal autopsies in separate groups.

### Ethical consideration

This study was approved by Ethical Review Committee of Aga Khan University and institutional review board (IRB) of WHO. Individual written informed consent was sought from each verbal autopsy respondent before entry into the study. Confidentiality of data was maintained throughout the study and was only accessible to the senior project staff. Participants in the study were allocated unique ID number. 

### Quality assurance

The quality was ensured by weekly review meetings and supervisory field visits. Random field visits were done by WHO member, the funding body, to ensure adequacy of procedures both in hospital and field. The verbal autopsy interview forms were double checked for completeness by supervisor before data entry. A random 5% of verbal autopsy interviews were also attended by the study supervisor. Compliance checks were done be once daily visit of the supervisor for validity of data. Daily progress report was generated and any problems faced were discussed with supervisor. 2% work of each field interviewer was verified by approaching the respondents directly by Social Scientist and Supervisor. Scheduled and random unscheduled visits for observation of fieldwork procedures and independent blind re-interviews were also conducted. 

### Data management & Analysis

Data was processed using the Visual FoxPro data management software (Fox Pro v 6.0 Microsoft Corp Seattle WA USA). Data entry was done using a standardized database structure. The database and range and consistency checks were prepared centrally with inputs from all sites. Range and internal consistency checks were performed regularly. The outcome measures were sensitivity and specificity of the verbal autopsy in ascertaining cause of death and cause specific mortality fractions. Verbal autopsy diagnoses were compared with the reference diagnoses using simple chi sq analyses. Sensitivity +10% precision and specificity +5% precision determined compared to the reference standard for all diseases. 

The chance corrected level of agreement between reference diagnoses and verbal autopsy was assessed using an inter-ratter agreement Kappa statistics (Cohen, 1960) with 95% confidence interval. Based on criteria originally proposed by Landis and Koch (25) kappa *K* value over 0.75 were taken as excellent agreement ,between 0.4 - 0.75 as moderate agreement , 0.21- 0.4 as fair agreement and below 0.2 as poor agreement .

## Results

Data was collected over 18 months from August 2006 to February 2008. Total 315 still births were recruited 13 did not gave consent and 20 resided in remote areas and thus not included in the study. 83 cases were excluded on the basis of shifting, wrong address or the home being locked “Figure 1”.

During study time 204 causes of still birth were identified in hospitals settings thus 204 hospital records were received by reviewer 1 and reviewer 2 and in case of discrepancy the assigned expert reviewed the case. Consensus observed between both reviewers for hospital record was 75.5% and 50 cases were discrepant, which were reviewed by expert similarly 60 cases were discrepant in VA forms thus causes of death similar in hospitals record and verbal autopsy was 46.1% [Table 1]. 

**Table 1:** Summary of reviewed cases of stillbirth.

	**Hospital Record**	**Verbal autopsy**
Reviewed cases by both reviewers	204	204
Consensus observed between both reviewers	75.5%	70.6%
Discrepant cases reviewed by third reviewer and finalized	50	60
Causes of stillbirth similar in hospital record and verbal autopsy	46.1%	

Table 2 shows baseline characteristics. Mean maternal age was 28 years. Most of the mothers were found to be anemic, Hemoglobin- 9.9 gm which depicts nutritional deprivation and poor socioeconomic status of the family. The mean gestational age and birth weight of these still births were 35.7 (+SD) weeks and 2.6 kg (+SD).

**Table 2:** Baseline characteristics of stillbirths.

**Characteristics of mother/stillborn**	**Stillbirths [N=204]**
Age of the mother (years), mean [SD]	28.6 [5.4]
Antenatal visits made, n [%]	83 [42.8]
Hemoglobin of mother (g/dl), mean [SD]	9.0 [2.0]
Gestation age (weeks), mean [SD]	35.7 [3.3]
Birth weight (grams), mean [SD]	2658.8 [952.8]
Lethal Congenital anomaly, n [%]	13 [6.7]
Multiple births, n [%]	6 [3.1]
Main respondent [verbal autopsy]	[N=204]
Age (years), mean [SD]	28.2 [6.1]
Education (years), mean [SD]	8.0 [2.9]

**Figure 5:**
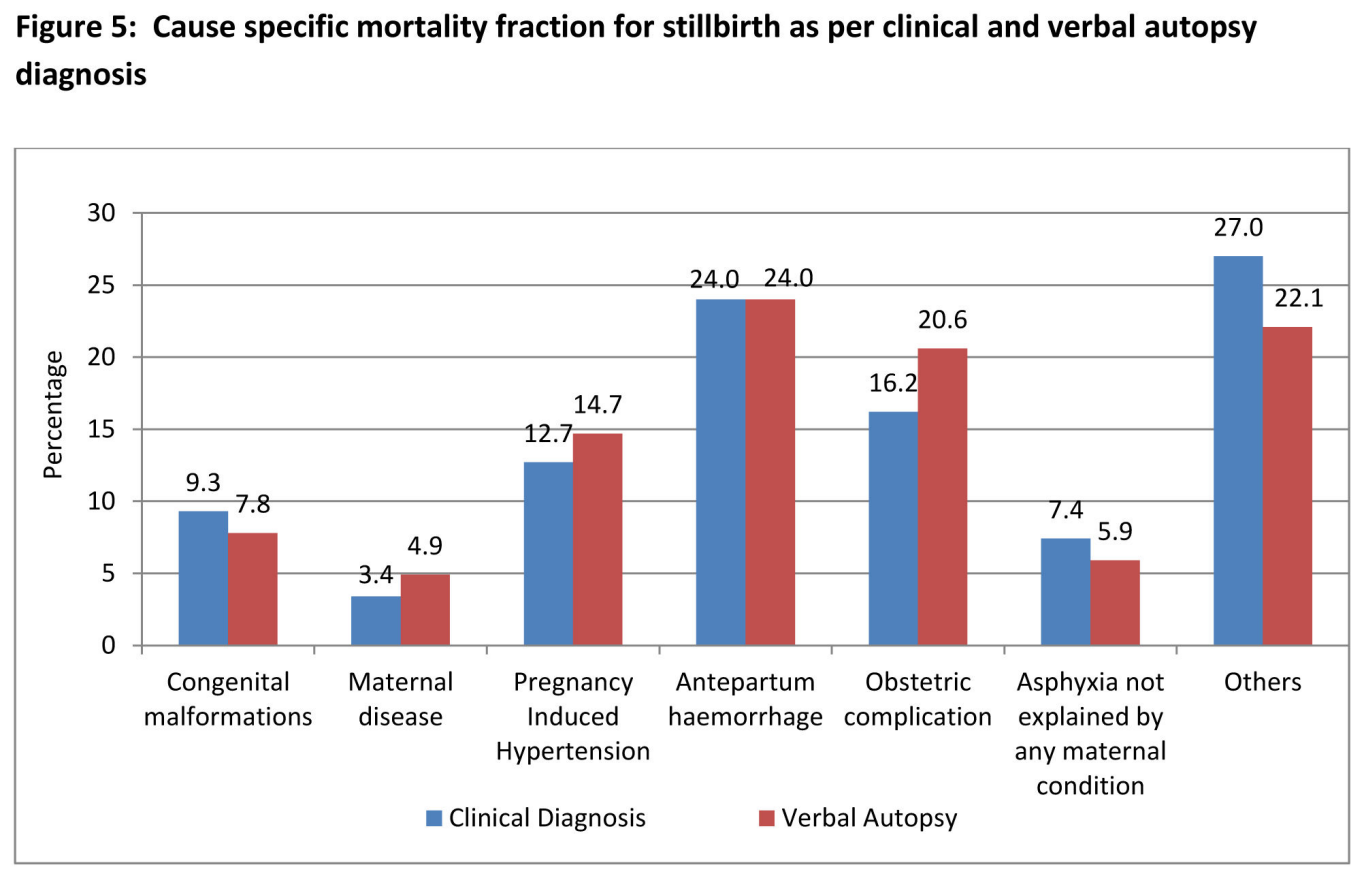
Cause specific mortality fraction for stillbirth as per clinical and verbal autopsy diagnosis.

Cause specific mortality fractions of the births were compared between hospital records and verbal autopsy [table 3]. Nearly 75% of these still births occurred due to antenatal complications, however intrapartum accidents were observed less frequent. Antepartum hemorrhage was seen in 24% of cases in both verbal autopsy and hospital records. Pregnancy induced hypertension was found in 12% in hospital records and 14% in verbal autopsy. Other leading causes included obstetric complications, congenital malformations and maternal diseases. Unexplained antepartum deaths were only 7.4% in hospital records. Leading cause of still birth in our study is Antepartum hemorrhage (24%) followed by obstetric complications (16.2% for hospital record and 20.6% for verbal autopsy), “Figure 5”. 

**Table 3:** Cause specific mortality fraction for stillbirth as per clinical verbal autopsy diagnosis.

**Causes of stillbirth**	**Clinical Diagnosis, n (%**)** [N=204**]	**Verbal Autopsy, n (%**)** [N=204**],	**KAPPA**	**P-value**
**Antepartum**	156 (76.5)	150 (73.5)	0.295	<0.001
**Intrapartum**	48 (23.5)	54 (26.5)		
Congenital malformations	19 (9.3)	16 (7.8)	0.72	<0.001
Maternal disease	7 (3.4)	10 (4.9)	0.45	<0.001
Pregnancy Induced Hypertension	26 (12.7)	30 (14.7)	0.34	<0.001
Antepartum haemorrhage	49 (24)	49 (24)	0.41	<0.001
Obstetric complication	33 (16.2)	42 (20.6)	0.4	<0.001
Asphyxia not explained by any maternal condition	15 (7.4)	12 (5.9)	0.09	0.02
Others	55 (27)	45 (22.1)	0.31	<0.001

### Diagnostic accuracy

Out of 204 still births, 80.8 % of ante partum and 50.5 % of intra partum deaths were correctly diagnosed by VA, however the specificity for ante partum and intra partum death was 50% and 80.8% respectively (table 4). Sensitivity of verbal autopsy is highest 68.4% for diagnosing congenital malformation followed by obstetric complications (57.6%); maternal diseases 57.1% and Antepartum hemorrhage 55.1%. 

**Table 4:** Sensitivity and Specificity of verbal autopsy against clinical diagnosis (hospital record).

**Cause of still birth**	**Sensitivity**		**Specificity**	
	**n/N**	**% [ 95% CI]**	**n/N**	**% [ 95% CI]**
**Antepartum**	126/156	80.8 [73.9,86.1]	24/48	50.0 [36.4,63.6]
**Intrapartum**	24/48	50.0 [36.4,63.6]	126/156	80.8 [73.9,86.1]
Congenital malformations	13/19	68.4 [46.0, 84.6]	182/185	98.4 [95.3, 99.4]
Maternal disease	4/7	57.1 [25.0, 84.2]	191/197	97.0 [93.5, 98.6]
Pregnancy Induced Hypertension	12/26	46.2 [28.7, 64.5]	160/178	89.9 [84.6, 93.5]
Antepartum haemorrhage	27/49	55.1 [41.3, 68.1]	133/155	85.8 [79.4, 90.4]
Obstetric complication	19/33	57.6 [40.8, 72.7]	148/171	86.5 [80.6, 90.8]
Asphyxia not explained by any maternal condition	2/15	13.3 [3.7, 37.8]	179/189	94.7 [90.5, 97.1]
Others	24/55	43.6[31.4,56.7]	128/149	85.9[79.4,90.6]

### Kappa

Level of agreement between reference cause of death and verbal autopsy was good for congenital malformation [kappa =0.72] and moderate for all major causes of still birth (k > 0.40) [Table 3]. There was lower accuracy and level of agreement for birth asphyxia and hypertension as a cause of still birth, which may be due to difficulties in providing precise description of these causes by the mother. 

## Discussion

This study is one of the largest validations of verbal autopsy for still birth in the region. We are aware of only two community based studies in resource limited settings, for diagnostic accuracy of verbal autopsy against reference standard cause of still birth; those are from rural Ghana [14] and Chandigarh, India [24]. Two other still birth diagnostic accuracy studies [15,16] are reported but they examined vital registration data and are from developed countries. Currently over 35 Demographic Surveillance Sites (DSS) in 18 countries, the Sample Registration System(SRS) sites in India and Disease Surveillance Points(DSP) system in China regularly use VA on a large scale, primarily to assess the cause of death of a defined population [17,19].

The verbal autopsy tool in our study has shown a specificity of 80.8% and a sensitivity of 80% for ante partum death. The specificity of most of the causes of death in our study being more than 90% is consistent with literature, which reports diagnostic accuracy of verbal autopsy to be acceptable at the individual level if the sensitivity and specificity are at least 90%. At the population level, the Verbal autopsy is deemed to have reasonable diagnostic accuracy if sensitivity is at least 50%, specificity at least 90% and CSMF is within 20% of the true value [23]. 

High sensitivity and specificity for congenital malformation 98.4%, in our study is consistent with results from Chandigarh [24]. However, it is contrary to what is reported earlier from rural Ghana [14]. One explanation could be the extensive training which was given to CHWs for digging out diagnoses and inclusion of supportive radiologic and laboratory data in assigning the cause of death .Secondly the verbal autopsy interviews within 6 weeks and preferably by mother has further reduced the chances of error.

Another, strength of our study is the minimization of unexplained deaths by using standard case definitions for assigning cause of death, extensive training of staff, doctors and reviewing by 2^nd^ and 3rd reviewer. Similar results are shown by Aggarwal [24] from Chandigarh. However previous studies from Ghana [14,15] reported 58-60% of unexplained ante partum deaths.

Cause specific mortality fractions found in the study are useful for strategic planning in both maternal and neonatal health care programs. High antepartum still birth rate (75%) than intrapartum (25%) is consistent with world literature which states about 2.2 million of stillbirths occur during last trimester but before the onset of labor [20], and also by Aggarwal [24]. Cause mortality fractions for stillbirth vary considerably in literature. We report leading cause of stillbirth as antepartum hemorrhage whereas multicenter study in low resource countries by Engmann C, et al [25] reported maternal and neonatal infections to be the major cause. Over two-thirds of the stillbirths are attributable to causes, for which preventive and therapeutic interventions are available, namely pregnancy-induced hypertension, antepartum hemorrhage, underlying maternal illness and obstetric complications. Interventions like better obstetric care, more rapid response to intrapartum complications, reducing delays at home and transportation should be integrated into antenatal and childbirth care. Secondly, the diagnostic accuracy of verbal autopsy suggests that the distribution of causes of death as determined by verbal autopsy can be confidently used to plan public health interventions. 

Reported literature on verbal autopsy vary markedly over the globe in terms of case definition, cause of death, classification system and reviewing verbal autopsy for assigning cause of death and this diversity makes it difficult to compare data [22]. In this regards WHO and its collaborators developed this modified verbal autopsy tool for neonatal death as well as stillbirths to identify the underlying causes of neonatal deaths and still birth, which has recently been used by Aggarwal [24]. We found it very effective, easy to use as the case definitions are simple to understand and applicable. It is for this reason, that the number of unexplained still births has been markedly reduced in our study.

There are limitations in this study. The reference cases were facility–based series in urban setting and may not be the representative of community as risks, exposure, interventions differ markedly .Therefore we report 16% of cases with obstetric complication whereas published studies from low income developing countries reports obstetric complication as leading cause of still birth[21] although the CSMFs were similar to community studies in West Africa[14] and other Global studies [2] .We used obstetricians reviews for assigning the cause of death which is the most commonly used method for assigning cause of death from verbal autopsy although the results vary considerably [7]. A disadvantage of this method is the lack of objectivity and inter-observer variability which we have addressed in our study by providing standard objective case definitions and hierarchy of causes of death to the physicians reviewing verbal autopsy interviews. Further, the method is labor intensive and is difficult to use in routine monitoring of causes of death, such as from Sample Registration Surveys in India and China [18,19]. An interesting alternative is the use of pre-decided computer algorithms. However, In spite of all limitations Quigley MA et al [23], strongly recommended physician reviews as it provided more accurate results in his study than application of computerized algorithms [23]. Obstetrician reviews in our study also limits its generalizability to other low-resource settings where obstetricians are unavailable and many births occur at home or other community settings. However, Engmaan C, et al [25] has recently used verbal autopsy interviews in non-hospital community based still births and early neonatal deaths in four low resource countries including Pakistan where cause assignment of death was done by two local physicians with reasonable sensitivity.

 Ascertainment of cause of still birth by two expert obstetricians, who worked independent and blind to each other and involvement of third obstetricians, in cases of discrepancy has decreased the bias as well as chances of error but this is an expensive approach and would be difficult to apply in community due to wide spread shortage of physicians in many low income countries. Verbal autopsy reviews by non-physicians after adequate training is therefore considered by many authors. However Engmann C, et al [26] reported an agreement of only 50% between physicians and non-physicians panels on ascertainment of cause of perinatal death. Thus further research is required before non-physicians are asked to determine perinatal cause of death in low income settings. 

## Conclusion

Our results suggest verbal autopsy tool as having reasonable validity in determining and discriminating between causes of stillbirth, thus can be used to estimate CSMFs of stillbirth at community level. However, as these validation results are hospital based care must be taken while interpreting data of still births that occur at home. Assignment of cause of death by obstetricians is an expensive and labour intensive method and can be replaced by general physician or non-physician in low income settings after further research .The introduction of uniform and reliable method to drive causes of death and standardization of the VA questionnaire and field operating procedures are important steps towards further improvement of the VA process. High antepartum deaths mostly due to antepartum hemorrhage and hypertension warrants public health interventions and allocation of appropriate resources to women in the immediate antenatal period to achieve Millennium Development Goals.
